# Identification of Unique Peptides for SARS-CoV-2 Diagnostics and Vaccine Development by an *In Silico* Proteomics Approach

**DOI:** 10.3389/fimmu.2021.725240

**Published:** 2021-09-24

**Authors:** Veerbhan Kesarwani, Rupal Gupta, Ramesh Raju Vetukuri, Sandeep Kumar Kushwaha, Sonu Gandhi

**Affiliations:** ^1^ DBT-National Institute of Animal Biotechnology (NIAB), Hyderabad, India; ^2^ Amity Institute of Biotechnology, Amity University, Mumbai, India; ^3^ Department of Plant Breeding, Swedish University of Agricultural Sciences, Alnarp, Sweden

**Keywords:** SARS-CoV-2, COVID-19 vaccines, diagnostic peptides, docking, paratopes, TCR, MHC

## Abstract

Ongoing evolution of severe acute respiratory syndrome coronavirus 2 (SARS-CoV-2) virus strains is posing new COVID-19 diagnosis and treatment challenges. To help efforts to meet these challenges we examined data acquired from proteomic analyses of human SARS-CoV-2-infected cell lines and samples from COVID-19 patients. Initially, 129 unique peptides were identified, which were rigorously evaluated for repeats, disorders, polymorphisms, antigenicity, immunogenicity, toxicity, allergens, sequence similarity to human proteins, and contributions from other potential cross-reacting pathogenic species or the human saliva microbiome. We also screened SARS-CoV-2-infected NBHE and A549 cell lines for presence of antigenic peptides, and identified paratope peptides from crystal structures of SARS-CoV-2 antigen-antibody complexes. We then selected four antigen peptides for docking with known viral unbound T-cell receptor (TCR), class I and II peptide major histocompatibility complex (pMHC), and identified paratope sequences. We also tested the paratope binding affinity of SARS-CoV T- and B-cell peptides that had been previously experimentally validated. The resultant antigenic peptides have high potential for generating SARS-CoV-2-specific antibodies, and the paratope peptides can be directly used to develop a COVID-19 diagnostics assay. The presented genomics and proteomics-based *in-silico* approaches have apparent utility for identifying new diagnostic peptides that could be used to fight SARS-CoV-2.

## Introduction

According to a World Health Organization report issued in May 2021, the SARS-CoV-2 virus has infected more than 158 million people, causing more than 3.3 million deaths worldwide (1, 2). Moreover, ongoing evolution of SARS-CoV-2 strains is posing constant challenges to develop new COVID-19 diagnoses and treatments for shifting life-threatening symptoms, *inter alia*, fever, respiratory distress, stomach ache and pneumonia (1–3). The SARS-CoV-2 virus has a 50–200 nm diameter and 27–30 Kb long single-stranded positive-sense RNA genome. This genome encodes large polyproteins (Orf 1a and 1b), four structural proteins (spike glycoprotein, envelope protein, membrane protein, and nucleocapsid protein), and five accessory proteins: Orf3a, Orf6, Orf7a, Orf8, and Orf10 (4). The spike protein is a key protein in host cell membrane attachment, as its S1 subunit binds to the human Angiotensin-Converting Enzyme 2 (ACE2 receptor) and activates the adhesion process (5, 6).

This involves a temporary hinge-like conformational movement of S1 receptor-Binding Domain (RBD) that enables binding to a protease domain (PD) of ACE2, which includes its alpha1-helix with inputs from its alpha2-helix and both β3 and β4 sheets (7, 8). Therefore, the spike protein appears to be the most suitable target for disease prevention, diagnosis, and treatment strategies.

Various molecular techniques such as Reverse Transcriptase Polymerase Chain Reaction (RT-PCR) analysis, Enzyme-Linked Immunosorbent Assays (ELISA), Western Blotting, Lateral Flow Immunoassays (LFIA), and Clustered Regularly Interspaced Short Palindromic Repeats (CRISPR)-based approaches have been used for SARS-CoV-2 diagnosis. However, these techniques are time-consuming, labor-intensive, and require substantial expertise. Currently, RT-PCR is widely considered the gold standard for confirmatory diagnosis (9–12). However, recent advances in proteomics have significantly contributed to disease diagnosis, elucidation of the host-pathogen interaction, disease biomarkers, antigens, and detection of antibodies in patient samples (13–17). Mass spectrometry (MS)-based proteomic methods have been used to detect SARS-CoV-2 viral proteins in human, animal, and cell line studies (*in-vitro* and *in-vivo*), and the virus at low loads in human samples (18, 19). In addition, targeted proteomic techniques have detected polypeptides of the SARS-CoV-2 nucleoprotein (20, 21), and several *in-silico* efforts have been made to identify antigenic peptides, T- and B-cell epitopes of SARS-CoV-2 proteins, and proteome sequences (22–25). Furthermore, transcriptomic studies have identified T- and B-cell epitopes (26) and the efficacy of the antiviral drug cepharanthine for COVID-19 treatment (27).

Since the pandemic began, numerous groups have studied COVID-19, generating enormous genomic and proteomic archives in the public domain. Therefore, we have developed a strategy, presented here, for identifying SARS-CoV-2 antigenic peptides and potential paratope peptides to detect viral antigens using publicly available resources. This involves an *in silico* approach for identifying and validating diagnostic peptides with the following steps. First, collection of genomic and MS-based proteomic data on the virus. Second, cataloging identified peptides’ antigenicity, immunogenicity, and toxicity. Third, selection of diagnostic peptides by removing potentially cross-reacting interfering peptides associated with human saliva and other pathogens. Fourth, verification of selected peptides’ expression in another infected cell line. Fifth, identification of paratopes for viral antigens. Finally, docking of the selected peptides with known viral TCR, class I and II pMHC, and the identified paratope peptides.

## Materials and Methods

### Collection of SARS-CoV-2 Virus Sequences to Explore Genomic Variability in the Spike and Nucleocapsid Proteins

All available SARS-CoV-2 spike and nucleocapsid nucleotide and protein sequences were extracted from the NCBI database using combinations of the keywords “COVID-19”, “SARS-CoV-2”, “spike,” and “nucleocapsid” both singly and in combinations with the Boolean operator AND. To generate a protein dataset, a local BLAST database was searched to find sequences with ≥ 95% similarity using protein sequences of Wuhan-Hu-1 isolates of SARS-CoV-2 (MN908947.3) as references. Sequences with non-standard amino acids were removed, and the remaining sequences were clustered using CD-HIT software with 100% sequence identity setting (28). To explore the genomic variability among the sequenced isolates, we applied multiple sequence alignment with ClustalW (29). Conserved and variable regions of the spike protein were identified using Gblocks software (30). To avoid selecting peptides with poor diagnostic potential, mutations in the protein detected in variants in all countries that had reported more than 10 spike protein sequences were analyzed. A binary matrix was generated for clustering based on the presence and absence of each identified mutation in the spike protein with respect to countries. This was done using the Clustvis web tool (31) and the following parameters. Clustering distance for rows and columns: binary. Clustering method for rows and columns: average. Tree ordering: tightest cluster first.

### Peptide Cataloging of the SARS-CoV-2 Proteome From Mass Spectrometric Proteome Data

The ProteomeXchange database was explored to extract SARS-CoV-2 mass spectrometric proteomic data using various keywords such as “SARS-CoV-2”, “COVID-19”, and “spike.” Two cell-line proteomes (PXD017710 and PXD018581) and four naturally infected patient proteomes (PXD019686, PXD021328, PXD018682, and PXD019423) were used to identify expressed SARS-CoV-2 peptides with Proteome Discoverer software (32–35). The extracted SARS-CoV-2 protein sequences and raw proteome files were the initial input for peptide identification with the following settings: 5% max. false discovery rate (FDR) at the protein level, at most one missed cleavage (1), 2–3 charge range (2–3), and 396–1,600 m/z range. A mass tolerance of 10 ppm was set for parent ions and 0.8 Da for fragment ions. The cell-line and patient sample proteomes were processed separately using human and virus reference sequences to explore differences between the two kinds of proteomes associated with infection by the virus.

### Network Analysis to Identify Hub and Bottleneck Genes

Immune system-related genes were identified to explore the protective immune response to infection by the virus in humans. A protein-interaction network analysis was constructed to identify key immune regulator genes among the identified proteins using the STRING 11.0 database with a threshold confidence score of 0.4 (36). The resulting interaction network was imported into Cytoscape 3.8.0 software for visualization. The Cytoscape plugin Cytohubba with an implemented 11-node ranking method was used to analyze the protein-interaction network. In addition, the degree of association and bottleneck approach was used to identify hubs and bottlenecks in the interaction network generated by the Network Analyzer plugin of Cytoscape (37).

### Filtering of Cross-Reacting Peptides

All the peptides in the generated catalogs similar to peptides of humans and other pathogens were removed to avoid misleading results from cross-reactive antibodies. Expressed human and human saliva microbiome peptides were extracted from The Human Protein Atlas (https://www.proteinatlas.org/) and proteomeXchange database (PXD003028), respectively. SARS-COV-2 peptides similar to peptides of host origin were filtered out using the phmmer program with default parameters (38). Peptides similar to those of pathogens inducing a clinical presentation similar to COVID-19, such as SARS-CoV, Influenza, Middle East Respiratory virus, Pneumoniae, Respiratory syncytial virus, Rhinovirus, *Staphylococcus aureus*, and *Streptococcu*s species in the Uniprot database were also filtered out using phmmer. The SARS-CoV-2 infected NHBE and A549 cell line proteomes were then explored for evidence of the selected peptides’ presence (26). Peptides expressed in all three experimentally generated data sources (cell lines, human patients, and proteome generated from cell-line RNA-Seq data) were retained for further study.

### Assessment of Antigenicity and Potential Immunogenicity of the Generated Peptides

In accordance with widely accepted definitions, the antigenicity of a peptide is regarded here as its capacity to bind specifically with a paratope, and its immunogenicity as its ability to induce an immune response, specifically production of antibodies against the antigenic protein (26). We used the Predicted Antigenic Peptides server (http://imed.med.ucm.es/Tools/antigenic.pl) to explore identified peptides’ antigenic potential and the Immune Epitope Database (IEDB) toolkit to explore their class-I pMHC immunogenicity (http://tools.iedb.org/immunogenicity), CD4 T-cell immunogenicity (http://tools.iedb.org/CD4episcore/), and binding to both class–I MHC (http://tools.iedb.org/mhci/), and class-II MHC (http://tools.iedb.org/mhcii/). A peptide inhibitory concentration (IC_50_) ≤ 900 nM was considered diagnostic of MHC class-I and II binding genes and alleles (39). B-cell epitopes for the spike protein RBD domain were identified using the Bepipred2.0 server with default parameter settings. All predicted epitopes were compared with those predicted by other tools for B-cell epitope prediction (BcePred, ABCpred, and SVM Trip) (40).

### Paratope Identification: Antigen-Binding Peptide Sequences

Complementarity determining regions (CDRs) are antibodies’ main antigen-binding domains, and most antigen-binding residues (ca. 80%) in paratopes are in CDR regions (41). To explore the SARS-CoV-2 antigen**-**binding peptide sequences, available crystal structures of antibody-antigen complexes involved in SARS-CoV-2 infection (PDB id: 7BWJ, 7BZ5, 7B3O, and 6W41) were downloaded from the RCSB Protein Data Bank (PDB) to extract light and heavy chain protein sequences. Paratome (42) and Parapred server (43) tools were used in conjunction with the extracted sequences to identify paratopes. Parapred applies a deep-learning architecture to integrate functionality from all local neighborhoods, while Paratome applies a machine learning approach based on multiple structure alignment (MSTA) of all available Ab-Ag complexes in the RCSB database. Only paratope sequences including sequences predicted by both tools were selected. The identified paratope peptides were assembled using the synthetic peptide linker GSGSGS to prevent undesired interactions between the discrete domains (44).

### Three-Dimensional Interaction Analysis of Selected Antigenic Peptides With Known Viral TCR, Class I and II MHC, and Paratope Peptides

Next, structural information on 19 well-known T-cell receptors (TCR) and 28 pMHC structures for different viruses were downloaded from the TCR3d database (45) for use in docking studies to assess the identified antigenic peptides’ structural compatibility with them. The antigen binding affinity of peptides of SARS-CoV-2 were identified by docking with selected paratopes of B cell and T-cell peptides (46, 47). 3D structures of B- and T-cell epitopes and those of the paratope peptides were predicted using the PEP-FOLD3 server (48). The identified SARS-CoV-2 peptides were docked with TCR and pMHC proteins using Cluspro 2.0, while paratopes were docked with the identified antigens, the independently predicted antigens of the RBD protein, and whole spike and RBD proteins using Cluspro 2.0 in antibody mode (49). Protein-paratope complexes were visualized and hydrogen bonds analyzed using the UCSF chimera (50) and LIGPLOT software (51).

## Results

**Figure 1:**
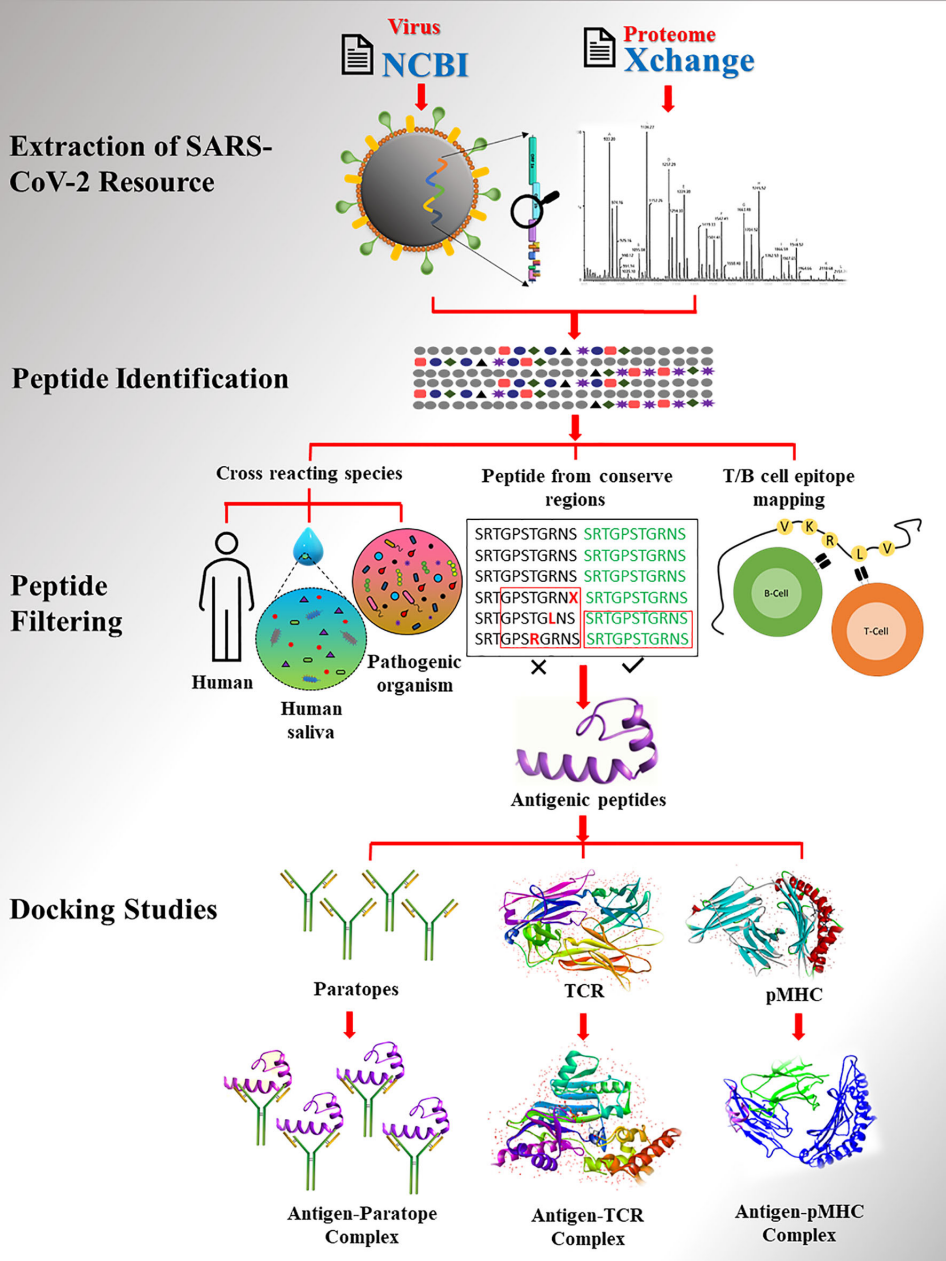
The workflow of study. SARS-CoV2 proteome extracted from database followed by peptide identification and filtering.

Numerous groups have studied the severity of COVID-19 since the pandemic began, resulting in massive genomics and proteomics resources in the public domain. Therefore, we have developed a strategic approach to identify unique SARS-CoV-2 antigenic peptides and potential paratope peptides to detect viral antigens using publicly available experimental resources. This involves a multi-step genomic and proteomic approach **(** **Figure 1** **)** for diagnostic peptide identification, and validation. Our study demonstrates a practical and precise approach for identifying diagnostic peptides when access to experimental sample data is limited. The identification of SARS-CoV-2 viral proteins highlights the value of today’s protein informatics resources in responses to a public health emergency.

### Meta-Analysis of the Genomic Diversity of the SARS-CoV-2 Virus

**Figure 2:**
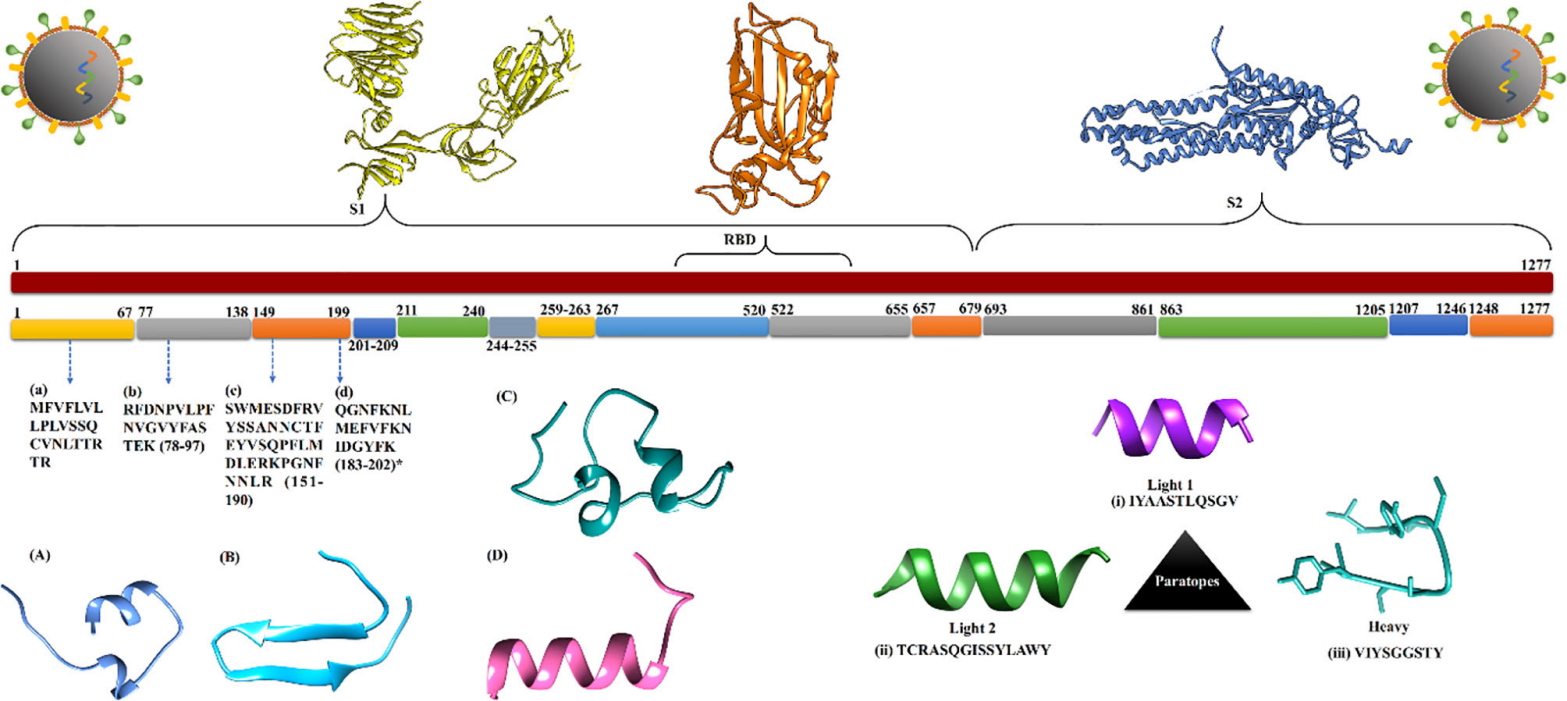
The conserved regions of spike protein extracted from protein data set of SARS-CoV-2. **(A–D)** Identified diagnostics peptide sequences with respect to their genomic location and 3D structure. **(i–iii)** Extracted paratopes sequence with their 3D structure.

Recently, various studies have reported genomic variation in SARS-CoV-2 viral strains and their severity. Thus, it is crucial to consider genomic variability when identifying and selecting peptides to develop robust diagnostic assays. To meet this need, we performed a large-scale meta-analysis of the variations in 358,558 protein sequences of SARS-CoV-2 detected in samples from 42 countries. A protein dataset was generated for each SASR-CoV-2 protein for sequence conservation analysis. We identified five regions [1–75, 79–197, 219–367, 374–390, and 398–423] and 14 regions [1–67, 77–138, 149–199, 201–209, 211–240, 244–255, 259–263, 267–520, 522–655, 657–679, 693–861, 863–1205, 1207–1246, and 1248–1277] for the nucleocapsid and spike proteins, respectively. Conserved regions of the spike protein are shown in **Figure 2**. In total, 149 spike mutations were identified in samples from all the countries. Mutation G614D, which increases transmissibility (52), was found in samples from 40 countries, while mutations F5L and F12S were found in samples from seven countries (Australia, Bahrain, Bangladesh, Canada, France, India, USA), and three countries (Egypt, Hong Kong, The Philippines), respectively. The numbers of protein sequences before and after clustering and heat map illustrating distributions of mutations in them are presented in **Supplementary File 1** **(** **Table S1**, **Figures S1**, **S2** **)**. Distribution of the mutations in countries and a binary matrix are provided in **Supplementary File 2** **(** **Tables S1**, **S2** **)**.

### SARS-CoV-2 Peptide Identification From Proteomic Data

Two cell lines and four naturally infected human patient proteomes were selected for the high-confidence identification of peptides using viral and human protein sequences as references. In total, 361 and 81 peptides of viral origin were identified in the cell lines and patient samples, respectively. Only three viral peptides in the cell-line and patient samples were identical. Analysis of the peptides revealed that they are encoded by various parts of the viral genome, such as the ORF1ab, nucleocapsid, envelope, and spike gene regions. Multiple peptides with varying lengths from different parts of the same proteins were found, including 57 component peptides of the spike protein. Of these 57 peptides, 28, 29, and one are components of the S1 (14–685), S2 (686–1273), and RBD (319–541) regions of the spike protein, respectively. The selected proteomes, samples, numbers of peptides, and identified viral proteins are briefly described in **Table 1** and **Supplementary File 2** **(** **Tables S3–S5** **)**.

**Table 1:** Summary of studied proteomes, number of uniquely identified peptides, and reference proteins.

Proteome Ids	Sample	Unique Peptide	Identified proteins	Proteome Ids
PXD017710	Colon Carcinoma-2 (Cell line)	148	ORF1ab, ORF3a, N, ORF10, S, ORF7a, ORF6, ORF1a, ORF8, ORF9b	PXD017710
PXD018581	H1299 (Cell line)	213	ORF10, ORF7a, N, ORF3a, ORF1ab, ORF8, S, ORF7b, ORF6, M, ORF6, NS, ORF1a, E	PXD018581
PXD021328	Naso and Oropharyngeal swabs (Human patient)	36	ORF10, ORF1ab, S, N, ORF7a, ORF3a, M	PXD021328
PXD019686	Nasal swab (Human patient)	25	ORF1ab, ORF10, S, N, ORF1a, ORF7a	PXD019686
PXD018682	Mouth Gargle (Human patient)	11	ORF10, ORF1ab, S	PXD018682
PXD019423	Mouth Gargle (Human patient)	9	ORF1ab, ORF3a, S	PXD019423

### Functional Analysis of the Proteomes From Infected Cell Lines and Samples From Naturally Infected Patients

Like any virus, SARS-CoV-2 must enter host cells and manipulate host responses to enable its replication. Therefore, exploration of protective immune responses to infection can provide important insights regarding viral pathogenesis. Thus, we explored host responses to the virus in both cell lines (Colon Carcinoma-2 and H1299) and naturally infected COVID-19 patients’ samples (mouth gargle, nasal swab, and respiratory tract). In total, 323 and 143 human peptides were identified in the cell line and patient samples, respectively. Only five (MDGA1, PIK3C2A, FOXP2, DCAF5, and IVD) were detected in both sets of samples. MDGA1 plays a role in formation or maintenance of inhibitory synapses (53), whereas PIK3C2A is involved in several intracellular trafficking and signaling pathways (54). FOXP2 is a transcription factor that may regulate hundreds of genes in several tissues, including the brain (55). DCAF5 is a receptor of CUL4-DDB1 E3 ubiquitin-protein ligase (56), and IVD is an essential enzyme for mitochondrial fatty acid beta-oxidation. Many of the other proteins are involved in immune system-related biological processes such as regulation of immune responses, autophagy, immune system development, leukocyte migration, antigen processing and presentation, or leukocyte-mediated cytotoxicity, and were detected in both cell line and naturally infected patient proteomes. Proteins involved in biological processes such as production of molecular mediators of immune response and myeloid cell homeostasis were only found in the cell-line proteome. As anticipated, peptides associated with the immune response and leukocyte activation were only found in the proteome of infected patients. In total, 58 and 23 unique genes related to immune system biological processes were found in the cell line and naturally infected patient proteomes, **(** **Supplementary File 3**: **Tables S1**–**S4** **)**.

**Figure 3:**
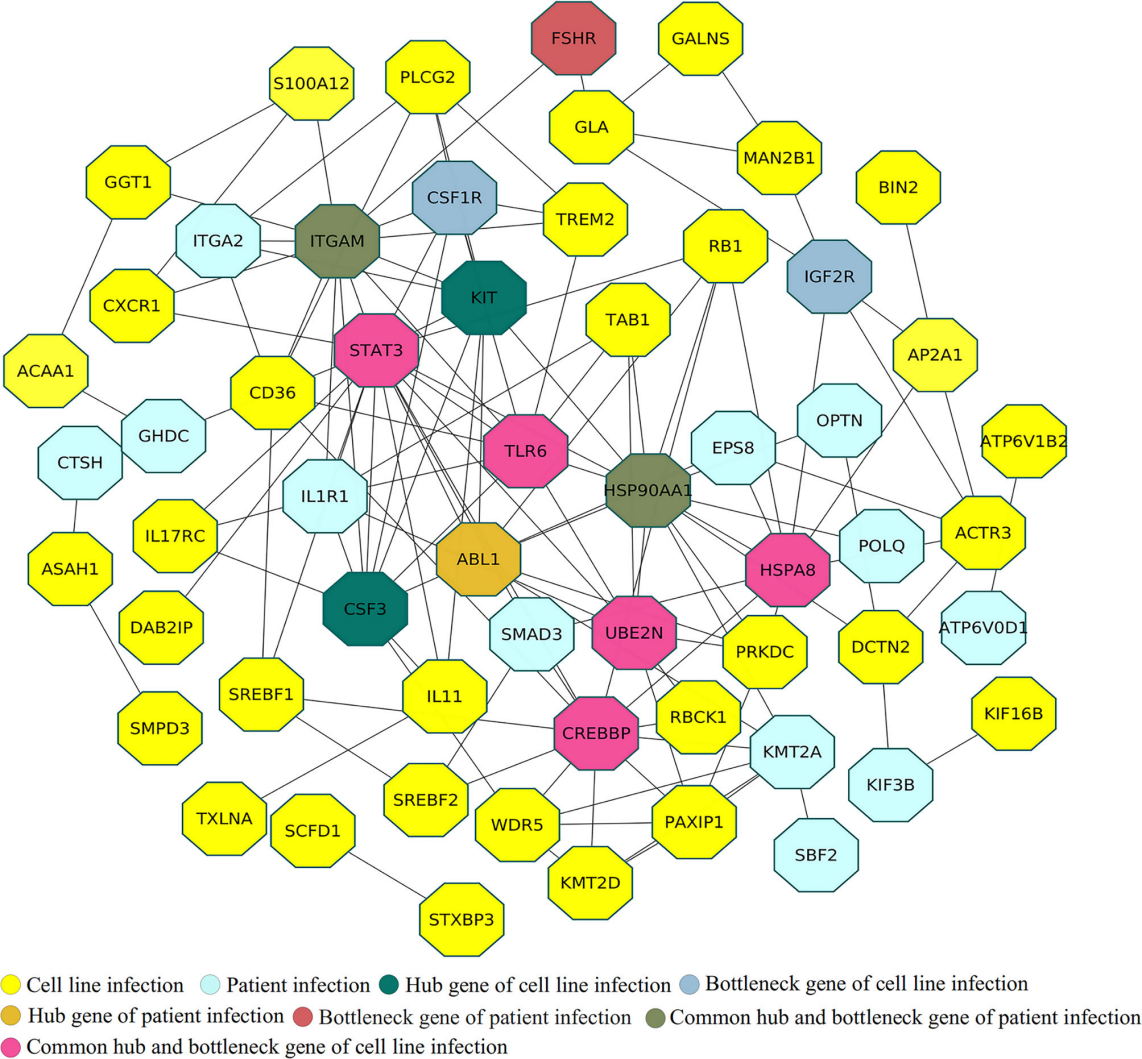
Protein interaction analysis among uniquely identified immune system genes in cell-line and patient proteomes.

The human innate immune system, which plays a crucial role in preventing infection and killing pathogens, involves various kinds of cells, including natural killer cells, macrophages, neutrophils, dendritic cells, and mast cells. Therefore, identifying proteins associated with both these cells and SARS-CoV-2 infection through analysis of experimental resources such as cell-line and patient datasets can improve understanding of interactions between the virus and human hosts. We found 33 innate proteins that matched entries in the InnateDB database. Most of these proteins are involved in immune-related functions such as protein binding (TAB1, SREBF2, HSP90AA1, RB1, STAT3, DCN, IL1R1, BNT3A2, PIK3R2, CCR6), transferase activity (TREM2, ABL1, S100A12, C4BPB), protein dimerization (UBE2N, CSF1R), and lipopeptide binding (EPS8, CD36). TAB1 may be involved in up-regulation of TAK1, IRF7, and IFN signaling during activation of the antiviral innate immune system (57). STAT3 has a well-known role in inflammation and immunity (58), and IL-1R signaling in CD4+ T-cells promotes Th17 immunity and atherosclerosis (59). TREM2 controls phagocytic pathways, which are involved in removal of neuronal debris (60). ABL1 is involved in regulating release of filoviruses through VP40 protein phosphorylation and might also be involved in the virus life cycle (61). EPS8 is a key regulator of the LPS-stimulated TLR4-MyD88 interaction and contributes to macrophage phagocytosis (62), while CD36 is a known scavenger receptor involved in immunity, metabolism, and angiogenesis (63). The major challenge was to identify key expressed immune genes in a complex network of the immune system. Therefore, the identified proteins related to the immune system process from cell-line and patient proteomes were used to generate a protein-interaction network **(** **Figure 3** **) (** **Supplementary File 1** **-** **Tables S2**, **S3** **)**. The generated protein-interaction network, which includes 403 nodes and 671 edges, was used to identify the top rank hubs and bottlenecks **(** **Supplementary File 1**, **Table S2** **)**.

### Selection of Diagnostic Peptides From the Generated Peptide Catalog

Antigenic peptides must, by definition, have sufficient antigenicity and immunogenicity to bind detectably to antigen-specific receptors on lymphocytes or the Fab region of antibodies. The antigenicity of a peptide is determined by surface epitopes of 5–7 amino acid residues, whereas four intrinsic properties of peptides determine their immunogenicity: chemical composition, molecular size, foreignness, and heterogenicity for processing and presentation on the surface of antigen-presenting cells (APCs). Therefore, we applied multi-step filtering to identify potential diagnostic peptides. Initially, to avoid future cross-reactivity, the identified peptides (442) were filtered to exclude human and human saliva microbiome peptides (418) and subsequently peptides of a targeted group of pathogenic bacteria and viruses (129). Next, to avoid selection of poor peptides for diagnostic purposes, the selected peptides’ expression was checked, using results of the infected cell-lines RNA-Seq data analysis. Finally, four peptides (**Table 2** and **Figure 2**), present in the NHBE and A549 cell lines, infected patient samples, and the RNA-Seq-derived proteome were selected after conservation analysis (**Supplementary File 4**: **Tables S1**–**S3**, and **S7**). A sequence alignment of all matched peptides from the three types of sources is provided in **Figure S3** of **Supplementary File 1**.

**Table 2:** List of selected peptides for SARS-CoV-2 diagnosis.

Data sources	Peptide ids	Length	Sequence	Protein accessions	Peptide positions
CL+NI+RNA	A26	40	SWMESDFRVYSSANNCTFEYVSQPFLMDLERKPGNFNNLR	QOD59281.1	S-S1 [151-190]
CL+RNA	A349	20	RFDNPVLPFNVGVYFASTEK	QOT61311.1	S-S1 [78-97]
	A194	23	MFVFLVLLPLVSSQCVNLTTRTR	QPG83544.1	S-S1 [1-23]
	A343	20	QGNFKNLMEFVFKNIDGYFK	QOI61545.1	S-S1 [183-202]

CL, Cell-line infected proteome; NI, Natural infected human patients; RNA, Transcriptome; S, Spike.

MHC genes, containing a set of closely linked polymorphic genes, encode crucial cell surface proteins that bind antigens, thereby alerting the immune system. Therefore, we evaluated the identified peptides’ antigenicity and CD4 immunogenicity to enable potency-based selection (**Table 3**). The average immunogenicity and antigenicity scores of peptides were approximately 89.06 and 1, respectively, which clearly showed the potential of selected peptides.

**Table 3:** CD4 immunogenicity and antigenicity of selected peptides.

Peptide id	Peptide Sequence	Peptide core	Immunogenicity Score	Combined Score
**CD4 Immunogenicity**				
A26	SWMESDFRVYSSANN	FRVYSSANN	91.1108	49.64432
A26	DFRVYSSANNCTFEY	FRVYSSANN	82.9956	46.39824
A194	MFVFLVLLPLVSSQCVNLTTRTR	FNVGVYFAS	91.22	48.125
A349	RFDNPVLPFNVGVYFASTEK	LLPLVSSQC	83.4206	47.64304
A343	NLMEFVFKNIDGYFK	FKNIDGYFK	77.9889	42.59556
**Antigenicity**				
**Peptide id**	**Peptide Sequence**	**Antigenic motifs**	**Length**	**Antigenic propensity**
A26	SWMESDFRVYSSANNCTFEYVSQPFLMDLERKPGNFNNLR	DFRVYSS, TFEYVSQPFLM	40	0.9981
A194	MFVFLVLLPLVSSQCVNLTTRTR	FLVLLPLVSSQCVNL	23	1.1110
A349	RFDNPVLPFNVGVYFASTEK	NPVLPFNVGVYFA	20	1.0446
A343	QGNFKNLMEFVFKNIDGYFK	LMEFVFK	20	0.9867
**B-cell linear epitopes**				
**Peptide id**	**Peptide Sequence**	**Epitope core**	**Length**	
A26	SWMESDFRVYSSANNCTFEYVSQPFLMDLERKPGNFNNLR	FRVYSSANN, DLERKPGNFN	40	
A194	MFVFLVLLPLVSSQCVNLTTRTR	RF	23	
A349	RFDNPVLPFNVGVYFASTEK	LVSSQCVNLT	20	
A343	QGNFKNLMEFVFKNIDGYFK	QG	20	

Class I and II MHC molecules have small grooves that present self-antigens and pathogen-derived peptides. Members of class I present intracellular antigens such as viruses, intracellular bacteria, or parasites to T cells, whereas the MHC class II presents exogenous antigens to professional APC, including lymphocytes, macrophages, dendritic cells, and Langerhans cells. Hence, information on MHC alleles’ binding of foreign peptide is crucial. The class I and II MHC alleles were evaluated by screening IEDB entries associated with the selected virus peptides. In MHC-I allele analysis, HLA-A and B type alleles were found to be the most frequently occurring (HLA-B*35:01; HLA-B*53:01; HLA-B*40:01; HLA-A*11:01; HLA-A*03:01; HLA-A*24:02; HLA-A*26:01; HLA-A*26:01; HLA-A*23:01; HLA-B*35:01; HLA-B*35:01; HLA-A*01:01; HLA-A*24:02; HLA-B*51:01; HLA-A*03:01; HLA-B*51:01; HLA-A*23:01; HLA-A*30:02; HLA-A*24:02; HLA-B*15:01; HLA-A*01:01; HLA-A*23:01; HLA-A*11:01; HLA-B*53:01). Similarly, MHC-II gene alleles were explored using the stabilized matrix-based method (SMM in IEDB analysis resources (**Supplementary File 4**: **Tables S4–S6**). The identified MHC class-II alleles for peptides are very common for exogenous antigens (HLA-DQA1*05:01, DQB1*03:01, HLA-DPA1*02:01, HLA-DRB1*04:01, HLA-DRB4*01:01) (64).

### Paratope Identification for Selected Peptides

Paratopes, sequences of 5–10 amino acids on antibodies that bind specific antigens, are preset at the three CDR regions (CDR1, CDR2, and CDR3), which are thus key regions for paratope identification. Two light chain (L1 and L2: IYAASTLQSGV and TCRASQGISSYLAWY, respectively) and one heavy chain (H: VIYSGGSTY) paratope sequences were identified using two prediction approaches **(** **Supplementary File 5**, **Table S3** **)**. The 3D structure of all three paratopes is shown in **Figure 2**. To increase the specificity of paratope sequences for the SARS-CoV-2 antigen, three paratope peptides were linked with a peptide linker (GSGSGS) to ensure that each assembled paratope peptide could work protein-independently, thus reducing unspecific antigen binding. Therefore, the light chain paratope L1 and heavy chain paratope H were stitched at the N and C termini of the first linker, and the second linker was attached to the C terminus of the heavy chain paratope (H) and N terminus of light chain paratope L2. In addition, we used a glycine-serine dimer (GSGSGS) triplet to assemble paratope peptides (IYAASTLQSGVGSGSGSVIYSGGSTYGSGSGSTCRASQGISSYLAWY).

### Docking Analysis of Peptides With the TCR and MHC

**Figure 4:**
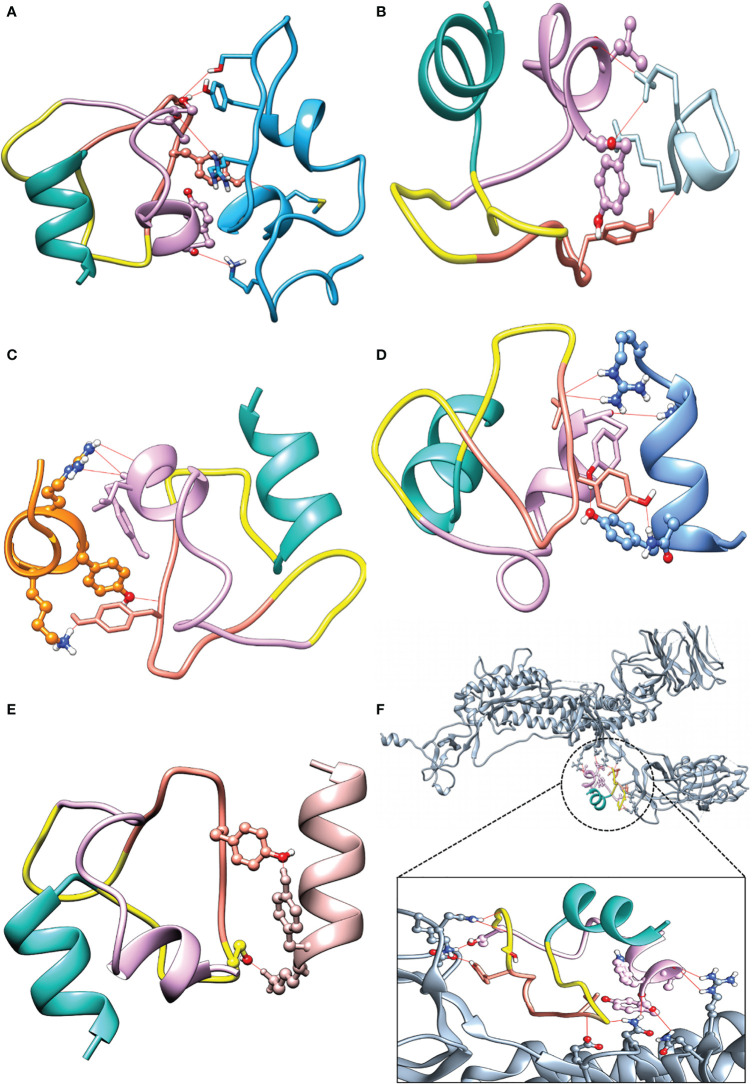
Docking complexes with assembled paratopes (AP) and its respective hydrogen bond numbers. **(A)** (AP - A26, 5). **(B)** (AP – SARS-CoV T-cell1, 3). **(C)** (AP – SARS-CoV T-cell2, 5). **(D)** (AP – SARS-CoV B-cell1, 5). **(E)** (AP – SARS-CoV B-cell2, 2). **(F)** (AP - whole spike, 10). AP color L1 (light sea green), L2 (Plum), H (salmon), and Linker (yellow).

Cellular immunity systems are activated once MHC molecules present endogenous or exogenous antigens at the cell surface to T cells. Therefore, we evaluated the affinity of well-known TCR receptors of viruses, class I and II MHC, and the identified paratope peptides, for the identified antigens in docking studies. Evaluation of docked complexes of the selected epitopes’ peptides A26, A194, A343, and A34919 with 19 MHC molecules and 28 TCR receptors yielded binding affinities ranging from -1138.9 to -741.4 and -1360.3 to -692.7 kcal/mol, respectively. A detailed description of all 19 TCR and 28 pMHC is provided in **Supplementary File 5**, **Tables S1**, **S2**. The molecular interaction of each antigen with each paratope was evaluated, the results are summarized in **Table 4**, and a detailed description is provided in **Supplementary File 5**, **Table S4**. The binding energies of paratopes for each antigen fell into three ranges (L1-antigens, -136.1 to -163.2 kcal/mol; L2-antigens, -199.9 to -242.3 kcal/mol; H-antigens, -181.6 to -202.6 kcal/mol) and all docked complex poses as well as the binding residues are presented in **Supplementary File 1**, **Figure S4**. The binding potential of the paratopes for the independently identified RBD antigens was also explored (**Supplementary File 5**, **Table S5**). To evaluate the assembled paratopes’ binding specificity, docking was done with each identified antigen, whole RBD, and spike protein, and the experimentally verified SARS-CoV T-cell and B-cell epitopes (derived binding energies: -196.8 to -235.2, -96.2 to -248, -303.3, -175.1 to -190.9 and -145.2 to -158.4 kcal/mol, respectively). The best-docked poses and several hydrogen bonds are shown in **Figure 4**. Our analysis indicates that the assembled paratope has strong binding affinity for the four identified antigens, RBD protein antigen, and whole spike protein. Moreover, the assembled paratope showed lower binding affinity for SARS-CoV T-cell (KCYGVSATKL, and NYNYKYRYLR) and B-cell (ISPYNTIVAKLR, and LSPLGALVACYK) epitopes.

**Table 4:** Antigen and paratope docking studies.

Antigens	Antigen-Paratope Binding energy	Hydrogen bonds	Interacting residues
A26	L1 (-145.8)	5	ILE1-PHE25, TYR2-PHE25, TYR2-GLU4, ALA3-GLN23, ARG40-THR6
	L2 (-242.3)	7	TYR11-LEU18, TRP14-HR22, TYR15-CYS15, TYR 15-MET1, TYR15-PHE2, TYR11-THR 19, TRP14-THR22
	H (-202.6)	3	TYR3-SER12, SER7-ASN15, THR8-ASN15
A194	L1(-163.2)	3	TYR2-LEU18,THR20-TYR2, THR20-TYR2
	L2 (-233.6)	3	TYR11-LEU18, TYR15-THR22, TYR11-THR22
	H (-190.1)	5	THR8-THR22, TYR9-VAL16, SER4-ARG21, TYR3-ARG21, GLY5-ARG23
A343	L1(-136.1)	3	TYR2-PHE10, GLY10-ASN3, SER9-ASN6
	L2 (-199.9)	3	ARG3-ASP16, ARG3-ASP16, TYR11-GLN1
	H (-181.6)	2	ASN14-TYR9, LYS20-TYR 3
A349	L1 (-156.9)	8	TYR2-ASN4, SER5-GLU19, SER5-GLU19, THR6-ARG1, ARG1-THR6, THR6- ARG1, TYR2-PHE2, GLY10-ASN10
	L2 (-219.6)	3	ARG3-ASP3, TYR11-ASN10, TYR11-ASN10
	H (-193.9)	5	TYR3-ASN4, SER7-ASP3, TYR9-PRO8, TYR9-ARG1, SER4-ASN4
**Docking studies of assembled paratopes (AP)**			
A26	AP (-235.2)	5	TYR 20-MET 3, SER 37-TYR 10, ALA 36-ARG 8, GLY 22-SER 12, TYR 47-LYS 32
A194	AP (-196.8)	7	ARG 35-GLN 14, ARG 35-GLN 14, TYR 47-THR 19, ALA 36-THR 19, TYR 20-ARG 21, VAL 18-ARG 21, VAL 18-ARG 21
A343	AP (-201.6)	4	TYR 20-MET 8, GLY 23-LYS 20, TRP 46-LYS 5, TYR 47-LYS 13
A349	AP (-211.2)	9	TYR 20-ASN 4, GLY 23-GLU 19, SER 37-THR 18, TYR 47-LYS 20, VAL 18-ARG 1, TYR 20-ARG 1, TYR 20-ARG 1, ALA 36-LYS 20, ILE 40-LYS 20
RBD	AP (-227.3)	7	TYR 2-TYR 449, GLY 22-GLY 485, ALA 45-TYR 449, SER 21-TYR 489, TYR 20-PHE 490, GLY 16-GLN 493, TRP 46-GLN 493
Spike	AP (-303.3)	11	ASN 556-SER 28, ASN 556-GLY 29, LYS 557-THR 33, ASN 616-GLY 16, ASN 616-TRP 46, GLN 644-TYR 47, ARG 646-LEU 44, ARG 646-LEU 44, GLU 619-VAL 18, ASP 574-TYR 26

## Discussion

RT-PCR is a widely accepted method for COVID-19 detection that involves sample collection, RNA extraction, reverse transcription, and targeted amplification of cDNA using appropriate primers for conserved regions: procedures that require high technical expertise. In addition to the long processing time (24–48 hours), RT-PCR also requires continuous monitoring of the genomic evolution of the virus to ensure that the primers are still valid. For these reasons, several COVID-19 diagnosis kits are available for testing. However, most kits lack field applicability, lack sufficient sensitivity, have long processing times, and provide undesirably high false-positive results. Globally, the number of cases is increasing due to various mutant strains. Therefore, fast, simple, and reliable diagnosis methods that can be applied used readily portable equipment are required for large-scale screening.

To assist efforts to develop such methods, we applied *in-silico* method techniques to identify unique SARS-CoV-2 peptides using experimentally generated data. The data explored in this study were originally generated with specific objectives. The PXD017710 cell line proteome was first used to identify drug targets and host cellular response players (34), the PXD018581 proteome was generated to compare SARS-CoV and SARS-CoV-2 virus disease progression, and the PXD021328, PXD019686, and PXD019423 proteomes were generated from infected human samples (mouth gargle, nasal and oral swab). Our primary objective was to develop robust, convenient, diagnostic methods for large-scale screening of human patient samples (20, 32, 33). However, none of the studies aligned with our aim and objectives. In this study, extracted virus protein and human proteome sequences were used to identify peptides from mass spectrometry data by exclusive data-processing flow (PWF_QE_Precursor_Quan and LFQ_MPS_SequestHT_Percolator). Several peptides of different lengths were identified from the whole genome of SARS-CoV-2 from both the cell line and patient proteomics data (**Table 1**). These results indicated that the trypsin digestion originally used was an appropriate choice for detecting viral peptides. To select mutation**-**free peptides, spike protein sequences were subjected to mutation analysis, and three major mutations (F5L, F12S, and G641D) were identified in samples from several countries. The G641D mutation, found in samples from all the countries, might be involved in viral conformational plasticity, increasing viral fitness (65). F5L and F12S mutations were also found in samples from several countries, but their impact on infection and disease progression is unclear. In a recent study (66), the E484K mutation was detected in a new variant (B.1.1.33) of the SARS-CoV-2 virus in Brazil. The E484K mutation has raised concern because it may increase the transmissibility of the virus. In our study, the E484K mutation was found in the Bahrain spike protein dataset.

The SARS-CoV-2 spike protein is one of the crucial targets for disease prevention, diagnosis and therapeutic antibody development. Its S1 region is responsible for binding to the host ACE2 receptor, and the S2 region is responsible for membrane fusion (67). Our results highlight four immunodominant SARS-CoV-2 peptides of the S1 region (A26, A349, A194, and A343**).** The identified peptides have high diagnostic potential due to appropriate proportions of hydrophilic residues (lysine, arginine, histidine, aspartic acid, glutamic acid, serine, threonine, tyrosine, asparagine, and glutamine) and immunogenic residues (lysine, arginine, glutamic acid, aspartic acid, glutamine, and asparagine), low number of internal cysteine residues, and absence of the arginine-glycine (RGD) tripeptide motif. Moreover, analysis of RNA-Seq data confirmed that the identified peptides are expressed in NHBE and A549 cells. The identified antigens were expressed in four cell lines (Colon Carcinoma-2, H1229, NHBE, and A549) and three types of human patient samples (mouth gargle, nasal, and oral swab) corroborating their expression in various cell types.

**Figure 5:**
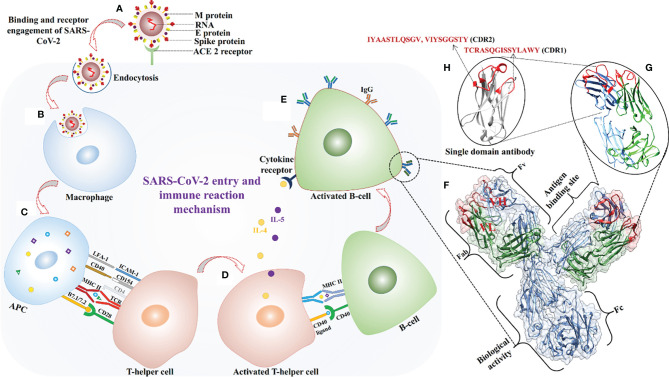
SARS-CoV-2 virus entry in the host cell and antigen-paratopes interaction. **(A)** Recognition of ACE-2 receptor and virus fusion. **(B)** Virus engulfment by a macrophage. **(C)** Antigens from digested SARS-CoV-2 proteins are presented with MHC-II on the cell surface and engagement of helper T-cell for its activation. **(D, E)** Activated helper T-cell interacted with B-cell *via* CD40 ligand and released cytokines for activation of B-cell for secretion of antibodies. **(F)** Enlarge view of antibody structure on the right side, heavy **(H)** and light (L) chains are shown in blue, and green respectively, CDRs are colored in red and light variable domains (V_H_ and V_L_) are labelled in red. **(G, H)** The identified paratopes of light (IYAASTLQSGV, TCRASQGISSYLAWY) and heavy (VIYSGGSTY) chain sequences are shown in red color.

The SARS-CoV-2 virus binds to the human ACE2 receptor through the spike protein’s RBD (**Figure 5A**), and enters cells *via* a mechanism involving a series of conformational changes in both viral and cell membrane proteins followed by an endocytic process. The identified expressed human genes reflect a protective immune response to SARS-CoV-2. Combined cell-line and human proteome data analysis captured immune proteins involved in different phases of the protective immune response, including antigen processing and presentation, and autophagy (**Figures 5B, C**). Various identified proteins such as DCTN2, KIF3B, and AP2A1 are involved in antigen processing for MHC class II molecules and the binding of antigen MHC-II complexes to TCR receptors (68, 69). Other proteins, like STAT3, ABL1, and IL1R1, help in the activation and multiplication of helper T-cells (**Figure 5D**) (70, 71). Activation of helper T-cells leads to B-cell activation and differentiation with OPTN, PLCG2, KLHL6, and TXLNA followed by production of B-cell antibodies (**Figure 5E**) (72, 73). Key immune hub and bottleneck genes were identified through protein-interaction network analysis. According to gene ontology analysis, most of the key genes are involved in host-virus interaction (*STAT3*, *CREBBP*, *HSPA8*, and *HSP90AA1*), innate immunity, T-cell differentiation, and the inflammatory response. (**Supplementary File 3**, **Tables S2**, **S3**).

Three paratopes [one heavy chain paratope from the CDR2 region (VIYSGGSTY), two light chain paratopes from CDR2 (IYAASTLQSGV), and a CDR1 paratope (TCRASQGISSYLAWY)] were identified from available X-ray crystallographic structures of antibodies (**Figures 5F–H**). Docking methods enable evaluation of the strength and nature of binding between biomolecules and hence validation of putative *in vitro* or *in vivo* interactions. Therefore, all four antigenic peptides were docked with 19 TCR receptors and MHC receptors, and the results clearly indicate that they had high binding affinity. Three paratope peptides were identified for diagnostic purposes, and most showed high binding affinity with all antigens. However, paratope L2 had the strongest binding affinity and formed several interacting hydrogen bonds (**Table 4**). To increase the diagnostic specificity for SARS-CoV-2 antigens, all the paratopes were then linked with commercially available glycine-serine-rich linkers. Docking studies showed that the designed paratope combination (IYAASTLQSGVGSGSGSVIYSGGSTYGSGSGSTCRASQGISSYLAWY) had stronger better binding affinity to different antigens and whole SARS-CoV-2 RBD and spike protein than the individual paratopes. The binding affinity of the assembled paratope peptide was also evaluated for experimentally validated B-and T-cell epitopes of SARS-CoV. The assembled paratopes showed higher binding affinity for SARS-CoV-2 antigens and proteins than for SARS-CoV. (**Supplementary File 5**, **Tables S5–S7**). Hence, the three identified paratopes and their assembled configuration with a glycine-serine rich linker were

## Conclusion

Various experimental and *in silico* efforts have provided valuable knowledge and resources (including massive genomic and proteomic datasets) to explore (*inter alia*) structural mechanisms of host-pathogen interactions, immune responses, drug candidates, antibodies, epitopes, genomic sequences and variation, infection rates, genome sequences. In this study we explored, available *in silico* resources, namely the cell-line and naturally infected COVID-19 patient’s proteomes, and identified four SARS-CoV-2 antigens and three antigen-binding peptides that could be used to develop diagnostic assays. The proposed antigenic peptides can be used for antibody generation, and the paratope sequences can be used directly for COVID-19 diagnostic assay and vaccine development. Moreover, the developed method and approaches can also be used to explore other infectious diseases

## Data Availability Statement

The RNA-Seq data used are available in the NCBI SRA database under project accession number PRJNA615032. Proteomic data are available in the ProteomeXchange database (cell-line proteomes PXD017710 and PXD018581; naturally infected patient proteomes PXD019686, PXD021328, PXD018682, and PXD019423).

## Author Contributions

SG conceptualized and provided overall guidance. SG, SK, RV, VB, and RG were involved in data curation, analysis, and interpretation. SK, SG, RV, VB, and RG wrote the manuscript. All authors contributed to the article and approved the submitted version.

## Funding

This work was supported by the Science and Engineering Research Board (SERB), New Delhi, *via* the Intensification of Research in High Priority Area (IRHPA) program (Grant Number IPA/2020/000069), FORMAS (2019-01316), and the Swedish Research Council (2019-04270)].

## Conflict of Interest

The authors declare that the research was conducted in the absence of any commercial or financial relationships that could be construed as a potential conflict of interest.

The reviewer JK declared a shared affiliation with one of the authors, RG, to the handling editor at the time of review.

## Publisher’s Note

All claims expressed in this article are solely those of the authors and do not necessarily represent those of their affiliated organizations, or those of the publisher, the editors and the reviewers. Any product that may be evaluated in this article, or claim that may be made by its manufacturer, is not guaranteed or endorsed by the publisher.
