# Supplementary material for: Identification of Unique Peptides for SARS-CoV-2 Diagnostics and Vaccine Development by an In Silico Proteomics Approach
Source: Front Immunol. 2021 Sep 24;12:725240. doi: 10.3389/fimmu.2021.725240 (PMC8498204; doi:10.3389/fimmu.2021.725240)
Supplement: Supplementary file 1 [file DataSheet_1.docx]

***In-silico* Proteomics Approach for Identification of Unique Peptides for SARS-CoV-2 Diagnostics and Vaccine Development**

Veerbhan Kesarwani^1^, Rupal Gupta^1,2^, Ramesh Raju Vetukuri^3^ Sandeep Kumar Kushwaha*^,1^, Sonu Gandhi*^,1^

^1^DBT-National Institute of Animal Biotechnology (NIAB), Hyderabad-500032, Telangana, India

^2^Amity Institute of Biotechnology, Amity University, Mumbai- 410206, Maharashtra, India

^3^ Department of Plant Breeding, Swedish University of Agricultural Sciences, Alnarp, SE-234 22, Sweden

*** Correspondence:**

Sandeep Kumar Kushwaha, Sonu Gandhi

[sandeep@niab.org](mailto:sandeep@niab.org), [gandhi@niab.org.in](mailto:gandhi@niab.org.in)

**Table S1 |** Number of extracted and filtered protein sequences for SARS-CoV-2 virus.

| Protein name | Sequence Count | Sequence count after the removal of non-standard amino-acid (X) | Sequence count after clustering |
| --- | --- | --- | --- |
| orf1ab | 39465 | 28156 | 6377 |
| orf1a | 39112 | 27123 | 5223 |
| Surface glycoprotein (S) | 40433 | 32274 | 1517 |
| orf3a | 81926 | 38949 | 751 |
| orf4 (E) | 40892 | 40600 | 73 |
| orf5 (M) | 40813 | 40130 | 207 |
| orf6 | 40990 | 40791 | 100 |
| orf7a | 37854 | 36756 | 294 |
| orf7b | 37589 | 37192 | 252 |
| orf8 | 40644 | 40065 | 343 |
| orf9 (N) | 40936 | 38485 | 981 |
| orf10 | 40784 | 40709 | 75 |
| Total | 521,438 | 441,230 | 16193 |

**
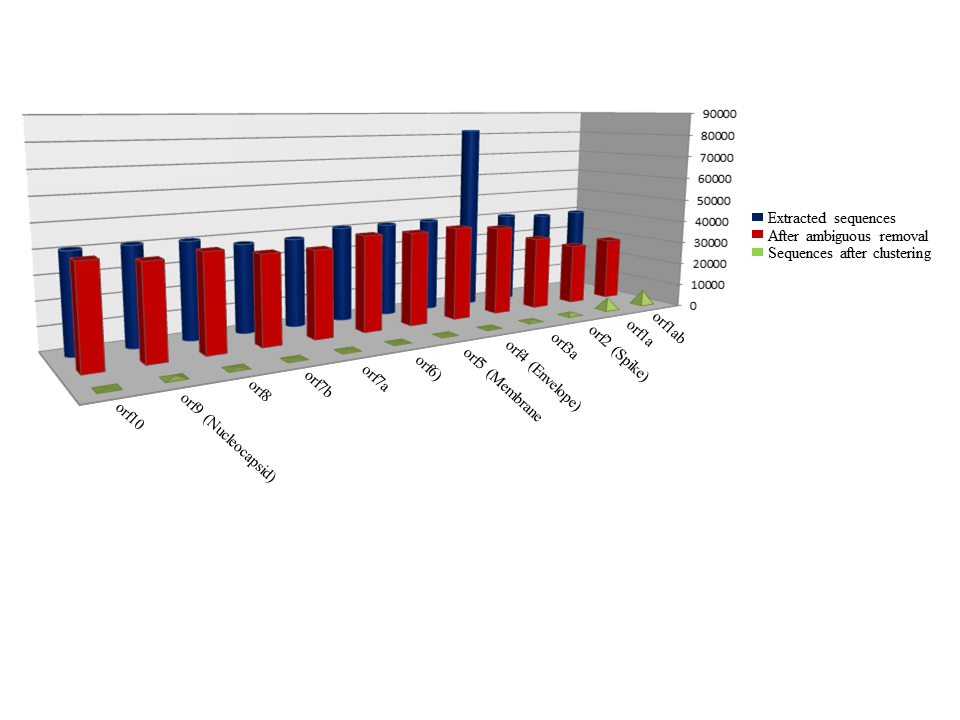
**

**FIGURE S1 |** Representation of table-S1: The number of unique protein sequences in public database. **Red:** number of extract protein sequences, **Blue:** number of protein sequences after clustering, **Green:** number of sequences after the removal of non-standard amino acids.


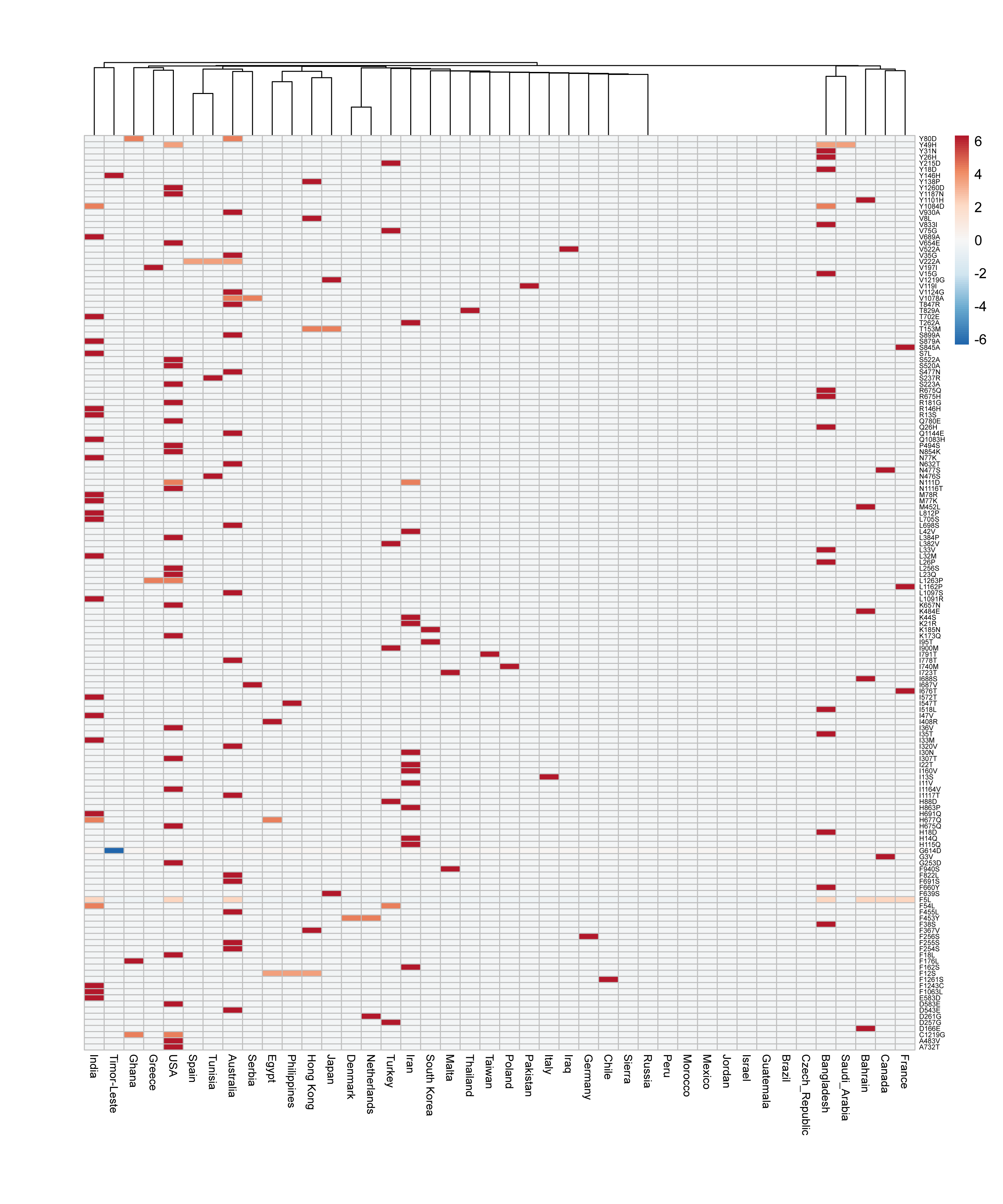
**FIGURE S2 |** Visualization of spike protein mutation with respect to countries through heat map. In heatmap, the column represented the countries and the rows corresponded to different mutation positions. The frequency of mutation on a logarithmic scale was encoded by a colored bar. Maximum mutation frequency values were displayed in red, and the smallest values were displayed in blue.

**Peptide 1 -** CL_26

CL_29 SWMESEFRVYSSANNCTFEYVSQPFLMDLEGR-------- 32

CL_15 SWMESEFRIYSSANNCTFEYVSQPFLMDLEGK-------- 32

CL_26 SWMESDFRVYSSANNCTFEYVSQPFLMDLERKPGNFNNLR 40

CL_25 SWMESEFRVYSSANNCTFEYVSQPFIMDLEGKQGNFK--- 37

NI_41 --------VYSSANNCTFEYVSQPFLMDLEGKQGNFKK-- 30

RNA_637 --------VYSSANNCTFEYVSQPFLMDLEGK-------- 24

RNA_834 --------VYSSANNCTFEYVSQPFLMDLEGKQGNFK--- 29

:****************:**** :

**Peptide 2** - CL_349

CL_349 RFDNPVLPFNVGVYFASTEK----- 20

RNA_744 -FDNPVLPFNDGVYFASTEKSNIIR 24

RNA_325 RFDNPVLPFNDGVYFASTEK----- 20

RNA_821 -FDNPVLPFNDGVYFASTEK----- 19

********* *********

**Peptide 3 -** CL_194

CL_194 --MFVFLVLLPLVSSQCVNLTTRTR 23

RNA_390 -TMFVFLVLLPLVSSQCVNLTTR-- 22

RNA_525 RTMFVFLVLLPLVSSQCVNLTTR-- 23

CL_224 --MFVFLVVLPLVSSQCVNLTTR-- 21

******:**************

**Peptide 4 -** CL_343

RNA_643 QGNFKNLR------------ 8

RNA_334 ---------NLREFVFK------- 8

CL_343 QGNFKNLMEFVFKNIDGYFK 20

RNA_20 ---------------------------NIDGYFK 7

RNA_31 -----------------EFVFKNIDGYFK 12

**FIGURE S3 |** Sequence alignment of four identified peptides sequences in cell-line, naturally infected human patients and proteome generated from RNA-Seq data of NHBE and A549 cell-line.


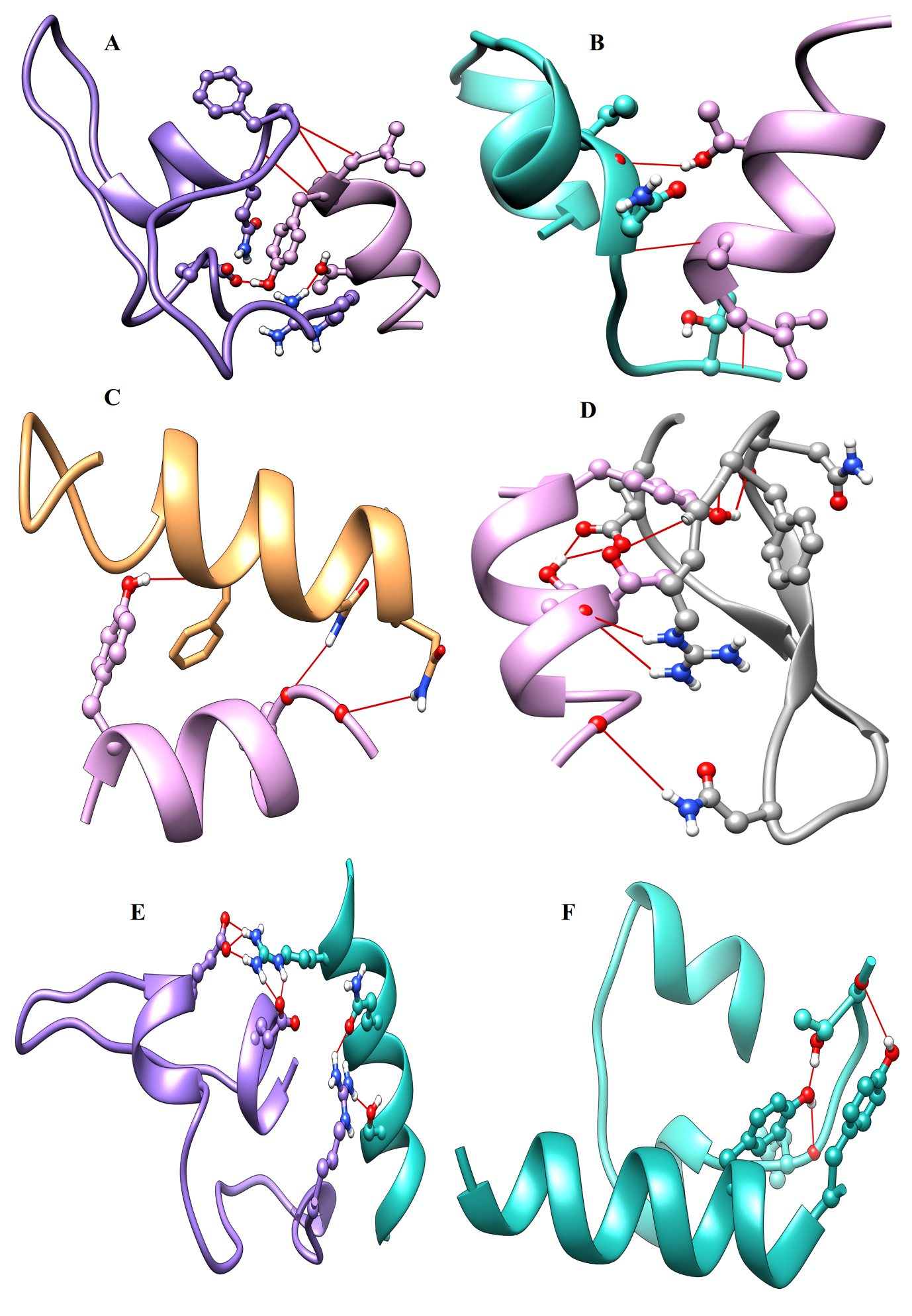


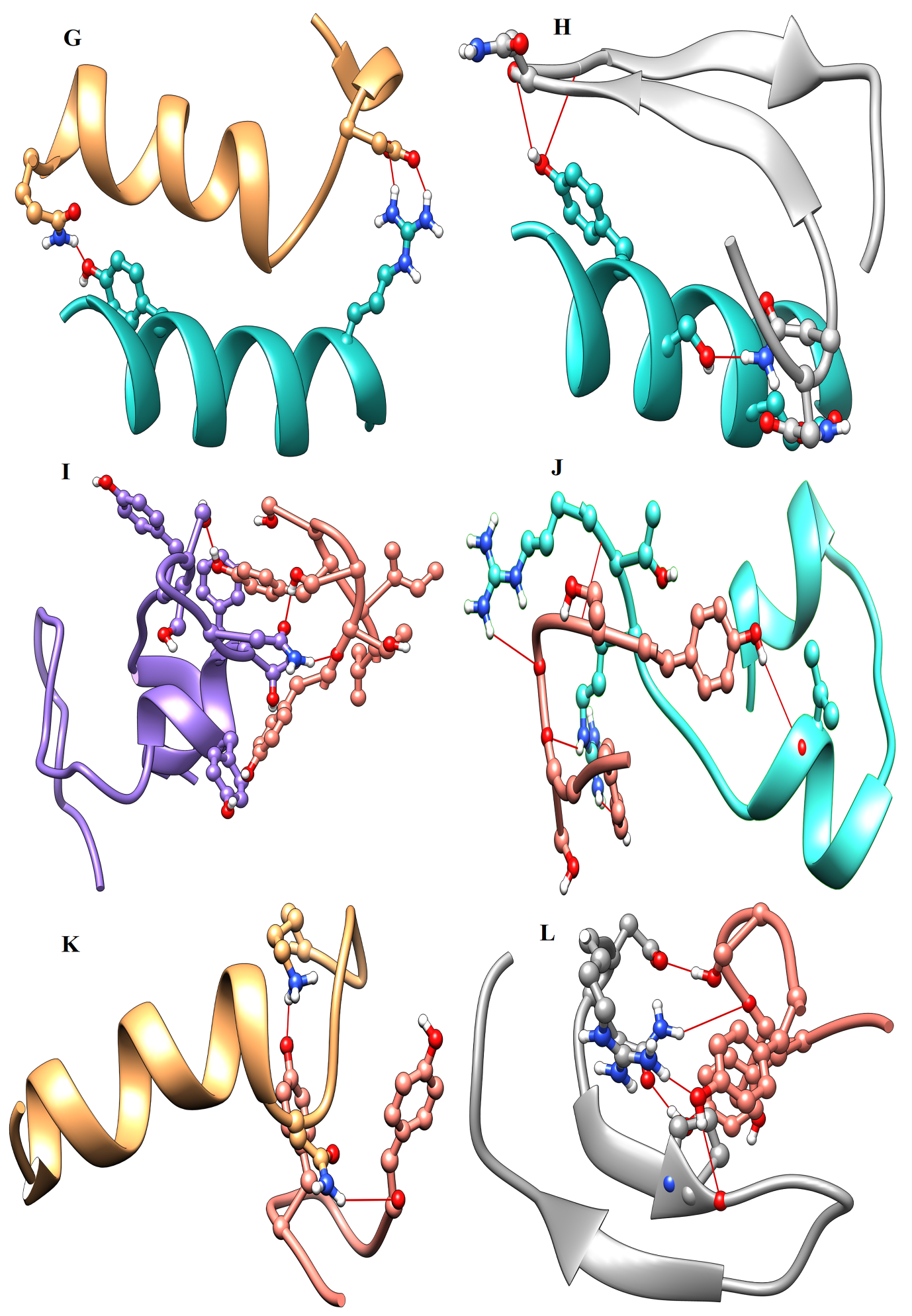


**FIGURE S4 |** Number of hydrogen between paratope (L1, L2 and H) and antigenic peptides (A26, A194, A343, A349). **A** L1-A26 (ILE1-PHE25, TYR2-PHE25, TYR2-GLU4, ALA3-GLN23, ARG40-THR6). **B** L1-A194 (TYR2-LEU18,THR20-TYR2, THR20-TYR2); **C** L1-A343 (TYR2- PHE10, GLY10-ASN3, SER9-ASN6), **D** L1-A349 (TYR2-ASN4, SER5-GLU19, SER5-GLU19, THR6-ARG1, ARG 1-THR6, THR6- ARG1, TYR2-PHE2, GLY10-ASN10), **E** L2-A26 (TYR11-LEU18, TRP14-HR22, TYR15-CYS15, TYR15-MET1, TYR15-PHE2, TYR11-THR19, TRP14-THR22), **F** L2-A194 (TYR11-LEU18, TYR15-THR22, TYR11-THR22), **G** L2-343 (ARG3-ASP16, ARG3-ASP16, TYR11-GLN1), **H** L2-349 (ARG3-ASP3, TYR11-ASN10, TYR11-ASN10), **I** H-A26 (TYR3-SER12, SER7-ASN15, THR8-ASN15), **J** H-A194 (THR8-THR22, TYR9-VAL16, SER4-ARG21, TYR3-ARG21, GLY5-ARG23), **K** H-A343 (ASN14-TYR9, LYS20- TYR3), **L** H-A349 (TYR3-ASN4, SER7-ASP3, TYR9-PRO8, TYR9-ARG1, SER4-ASN4)

**Table S2 |** List of top 10 hub and bottleneck genes identified from protein interaction network of immune genes.

| **Rank** | **EnsembleID** | **Gene (Degree)** | **Description** | **Tissue specificity** | **Biological process** |
| --- | --- | --- | --- | --- | --- |
| **Identified hub genes in immune gene interaction network** | | | | | |
| 1 | ENSG00000168610 | STAT3 (18) | Signal transducer and activator of transcription 3 | Low tissue specificity | Host-virus interaction, Transcription regulation |
| 2 | ENSG00000080824 | HSP90AA1 (13) | Heat shock protein 90 alpha family class A member 1 | Vagina | Host-virus interaction, Stress response |
| 3 | ENSG00000169896 | ITGAM (13) | Integrin subunit alpha M | Bone marrow | Cell adhesion, Immunity, Innate immunity |
| 4 | ENSG00000005339 | CREBBP (12) | CREB binding protein | Low tissue specificity | Biological rhythms, Host-virus interaction, Transcription regulation |
| 5 | ENSG00000108342 | CSF3 (10) | Colony stimulating factor 3 | Cervix, uterine, lung | Cellular response to cytokine stimulus, granulocyte differentiation, immune response |
| 6 | ENSG00000174130 | TLR6 (10) | Toll like receptor 6 | Blood, brain, lymphoid tissue | Inflammatory response, Innate immunity |
| 7 | ENSG00000157404 | KIT (9) | KIT proto-oncogene, receptor tyrosine kinase | Breast | T cell differentiation, Fc receptor signaling pathway, B cell differentiation |
| 8 | ENSG00000109971 | HSPA8 (9) | Heat shock protein family A (Hsp70) member 8 | Low tissue specificity | Host-virus interaction, mRNA processing, mRNA splicing, Stress response, Transcription regulation |
| 9 | ENSG00000177889 | UBE2N (8) | Ubiquitin conjugating enzyme E2 N | Low tissue specificity | DNA damage, DNA repair, Ubl conjugation pathway |
| 10 | ENSG00000097007 | ABL1 (8) | ABL proto-oncogene 1, non-receptor tyrosine kinase | Low tissue specificity | Apoptosis, Autophagy, Cell adhesion, DNA damage, DNA repair, Endocytosis |
| **Identified bottle-neck genes in immune gene interaction network** | | | | | |
| 1 | ENSG00000168610 | STAT3 (20) | Signal transducer and activator of transcription 3 | Low tissue specificity | Host-virus interaction, Transcription, regulation |
| 2 | ENSG00000080824 | HSP90AA1(12) | Heat shock protein 90 alpha family class A member 1 | Vagina | Host-virus interaction, Stress response |
| 3 | ENSG00000005339 | CREBBP (12) | CREB binding protein | Low tissue specificity | Biological rhythms, Host-virus interaction, Transcription, regulation |
| 4 | ENSG00000169896 | ITGAM (11) | Integrin subunit alpha M | Bone marrow | Cell adhesion, Immunity, Innate immunity |
| 5 | ENSG00000109971 | HSPA8 (9) | Heat shock protein family A (Hsp70) member 8 | Low tissue specificity | Host-virus interaction, mRNA processing, mRNA splicing, Stress response, Transcription, Transcription regulation |
| 6 | ENSG00000170820 | FSHR (4) | Follicle stimulating hormone receptor | Gallbladder, ovary, testis | Activation of adenylate cyclase activity, regulation of protein kinase A signaling, spermatogenesis |
| 7 | ENSG00000177889 | UBE2N (4) | Ubiquitin conjugating enzyme E2 N | Low tissue specificity | DNA damage & repair, Ubl conjugation pathway |
| 8 | ENSG00000197081 | IGF2R (4) | Insulin like growth factor 2 receptor | Low tissue specificity | Golgi apparatus, Vesicles |
| 9 | ENSG00000174130 | TLR6 (4) | Toll like receptor 6 | blood, brain, lymphoid tissue | Inflammatory response, Innate immunity |
| 10 | ENSG00000182578 | CSF1R (3) | Colony stimulating factor 1 receptor | Blood, lymphoid tissue | Inflammatory response, Innate immunity |

*Tissue specificity data of identified genes were extracted from human proteome atlas.
